# Effect of topical ropivacaine on the response to endotracheal tube during emergence from general anesthesia: a prospective randomized double-blind controlled study

**DOI:** 10.1186/s12871-018-0601-x

**Published:** 2018-09-27

**Authors:** Panpan Fang, Zhijun Zong, Yao Lu, Xiaoyu Han, Xuesheng Liu

**Affiliations:** Department of Anesthesiology, The First Affiliated Hospital of Anhui Medical University, Hefei, 230022 Anhui Province People’s Republic of China

**Keywords:** Ropivacaine; Anesthesia; Local; Anesthesia Recovery Period; Anesthesia and Analgesia; cough

## Abstract

**Background:**

The airway reflex such as cough is common accompanied with severe fluctuations of hemodynamics during emergence. This prospective double-blind randomized controlled trial tested the hypothesis that topical ropivacaine may reduce extubation response and postoperative sore throat.

**Methods:**

Fifty-four patients undergoing thyroidectomy were randomly assigned to two groups. The patients in Group R were received 0.75% ropivacaine, which was sprayed on the tracheal mucosa, epiglottis, tongue base, and glottis to achieve uniform surface anesthesia. As control, patients in Group C were received the same volume saline. The primiary outcome was the incidence and grade of cough during peri-extubation.

**Results:**

The incidence (34.62% vs. 76.92%, *P* = 0.002) of cough during extubation were lower in Group R compared to Group C. Meanwhile, the sore throat visual acuity score at 12 h after surgery was lower in Group R than that in Group C (2.00 vs. 3.50, *P* = 0.040).

**Conclusion:**

Topical anesthesia with 0.75% ropivacaine before intubation can significantly reduce the incidence of cough during peri-extubation. Meanwhile, it reduced hemodynamic fluctuations and postoperative throat pain without influence patients recovery.

**Trial registration:**

Chinese Clinical Trial Registry, ChiCTR1800014412 (date of registration January 2018).

## Background

Both intubation and extubation can cause acute hemodynamic changes [1]. Acute severe hemodynamic changes during extubation may lead to life-threatening complications [2, 3]. Opioids and lidocaine are often used to reduce intubation stimulation [4]. However, hazards in extubation response are often neglected. During emergence from general anesthesia, the incidence of cough is reported to be 67–80% in a mixed major surgical population [5, 6]. Various methods have been applied to attenuate extubation response during emergence, including alkalinized lidocaine in the endotracheal tube cuff [7]; laryngotracheal topicalization with lidocaine [8]; and IV administration of lidocaine, dexmedetomidine [9, 10], or remifentanil [11]. Concerns about the use of these techniques include delayed emergence from anesthesia, respiratory depression, sedative effects, postoperative nausea and vomiting (PONV), and short action time. Topical anesthesia with ropivacaine has been reported to attenuate extubation response in hypertensive surgical patients and by trans-cricothyroid membrane injection [12, 13]. But in genrenal population and with other mode of administration, the effect of topical ropivacaine anesthesia was not clear. The purpose of this study was to investigate whether topical ropivacaine anesthesia can increase the tolerance to the endotracheal tube to facilitate early and rapid recovery of surgical patients (enhanced recovery after surgery, ERAS) [14] post-thyroidectomy.

## Methods

### Participants

This study was prospective, patient and investigator blinded, controlled, parallel-group clinical trial with equal randomization, approved by the Ethics Committee of the First Affiliated Hospital of Anhui Medical University (approval number: PJ2017-12-13) and registered in the Chinese Clinical Trial Registry (ChiCTR1800014412). The study took place at the First Affiliated Hospital of Anhui Medical University.

Between February 2018 to May 2018, American Society of Anesthesiologists (ASA) I or II patients scheduled for elective thyroidectomy under general anesthesia were recruited. Exclusion criteria were severe cardiovascular, liver, and kidney disfunction; allergies to amide local anesthetics; difficult airway or history of maxillofacial and neck surgery; chronic respiratory disease such as chronic obstructive pulmonary disease or asthma, recent respiratory tract infection, chronic cough, and current smoking. Patients with reoperation because of serious adverse events such as bleeding, anesthesia time more than 4 h, and delayed extubation (more than 1 h without extubation) because of adverse effect to experimental drugs, transferd to the Intensive Care Unit (ICU) with the tube were eliminated. Written informed consent were obtained from all subjects.

Subjects were randomised to Group R or Group C with a 1:1 allocation using computer-generated random number. If the random number is odd, the patient will be allocated into Group R, or into Group C. Group assignments were sealed in sequentially numbered opaque envelopes, which were opened after the patients provided informed consent. The anesthesia nurses prepared the experimental drugs according to group assignments in syringes which has no difference in appearance; The patients, data collectors (anesthetist) did not know the drugs used for topical anesthesia.

### Study protocol

Heart rate (HR), blood pressure (BP), electrocardiograph (ECG), blood oxygen saturation (SpO_2_), end-tidal CO_2_ (EtCO2), and bispectral index (BIS) were routinely monitored in operation room. Before anesthesia induction, 0.5 mg of penehyclidine hydrochloride and 0.5 mg of dexamethasone were given intravenously.

Anesthesia induction was performed by a senior anesthesiologist. All patients received 0.02–0.06 mg/kg of midazolam, 0.4–0.6 μg/kg of sufentanil, 0.2–0.4 mg/kg of cisatracurium besilate and 0.2–0.4 mg/kg of etomidate for induction. When the muscles were completely relaxed and BIS dropped to 50 to 55, we used a laryngo-tracheal mucosal atomization device (Wolfe Tory Medical, Inc. Produced in State of Utah, USA) and 6 mL of drug (Group R: 0.75% ropivacaine; Group C: saline) to allow uniform surface anesthesia. The maximal tolerable dose of ropivacaine in human is 3 mg/kg. Drug distribution was as follows: under the glottis 3 ~ 4 cm (tracheal mucosa) spray: 2 mL, epiglottis and the tongue root spray: 2 mL, both sides of the glottis: 2 mL. Assisted breathing was performed via the face mask for 2 min before endotracheal intubation. The inner diameter of the endotracheal tube used was 7.0 mm for female patients and 7.5 mm for male patients. Cuff pressure was controlled at 20–25 mmHg and was monitored with a pressure gauge. Intubation was performed by two anesthesiologists within 30 s. After successful intubation, mechanical ventilation (tidal volume: 6–8 mL/kg, respiratory rate : 10–12 breaths/min, expiration:inspiration = 2:1) took over, and the end-tidal carbon dioxide pressure was maintained at 35–40 mmHg.

Anesthesia was maintained by 50–100 mcg/kg/min of propofol, 0.1–1 mcg/kg/min of remifentanil, and intravenous cisatracurium besilate every 30–50 min to maintain the suitable depth of anesthesia (BIS 40–60). We used a muscle relaxation detector to monitor muscle relaxation effects. At the end of surgery, 100 mg flurbiprofen was used for postoperative pain. When anesthesia was over, 0.01–0.02 mg/kg of neostigmine was administered to antagonize the non-depolarizing muscle relaxants and make T4/T1 > 0.9. Machine-controlled breathing was shifted to manual breathing with ETCO_2_ not exceeding 60 mmHg until the patients resumed spontaneous breathing. When the respiratory rate was greater than 10 breaths/min and tidal volume was more than 200 mL, the patients were transferred to the post-anesthesia care unit (PACU) for wakefulness observation and extubation. The endotracheal tube was pulled out when the following parameters were met: respiratory rate was 12–30 breaths/min, tidal volume was 6 mL/kg, SpO_2_ ≥ 95%, SpO_2_ ≥ 90% with respiratory air, and swallowing and cough reflex recovery were present. Patients with stable vital signs and Steward score ≥ 6 points were transferred to the general ward.

### Data collection

The anesthesiologist recorded the start time of anesthesia (the start time of the sufentanil bolus), start time of operation (start of skin incision), end time of operation time (sewing last needle), and end time of anesthesia (the time where propofol infusion was stopped). Systolic BP (SBP), diastolic BP (DBP), mean arterial pressure (MAP), and HR were recorded before anesthesia induction (T0), before intubation (T1), immediately after intubation (T2), 5 mins after intubation (T3), at the end of surgery (T4), immediately after extubation (T5), and 5 mins after extubation (T6).

In the PACU, the patients were observed for hypertension (more than 20% above the baseline or ≥ 160/95 mmHg) or agitation during recovery (assessed by the Richmond Agitation and Sedation Scale). We also recorded nausea and vomiting, grade of coughing (grade 0: no cough; grade 1: single cough with mild severity; grade 2: cough lasting less than 5 s with moderate severity; grade 3: more than 5 s of persistent cough), patient eye-opening time (from the end of anesthesia to the time where patients opened their eyes), time of extubation (from the end of anesthesia to the time the endotracheal tube was pulled out), and time spent in the PACU. Patients were followed up 12 h after surgery to record the degree of sore throat and incision pain (assessed by visual acuity scores(VAS)). We evaluated patients for residual adverse reactions from surface anesthesia including numbness in the throat, coughing when drinking water, hoarseness and tone down.

### Statistical analysis

Based on the published peri-extubation cough studies, we estimated the incidence of coughing to be between 67 and 80% in a mixed major surgical population. Our pilot experiment indicated that the incidence of peri-extubation coughing was 30% with local ropivacaine. Assuming a 5% two-tailed type I error rate, a sample size of 42 was needed to provide greater than 90% power to detect a decrease in the incidence of coughing from 73% in the control group (placebo) to 30% in the local ropivacaine group.

All data are expressed as mean (SD), median (range), or number (proportion, %). Data were analyzed using SPSS version 16.0 (SPSS Inc., Chicago, IL, USA). We used an unpaired two-tailed Student’s t-test or repeated measures analysis of variance with Bonferroni correction to compare normally distributed continuous variables. Continuous data that were not normally distributed were analyzed using the Mann–Whitney U-test. Categorical data were analyzed using the χ^2^ test or Fisher’s exact test where appropriate. A *P*-value < 0.05 was considered statistically significant.

## Results

**Fig. 1:**
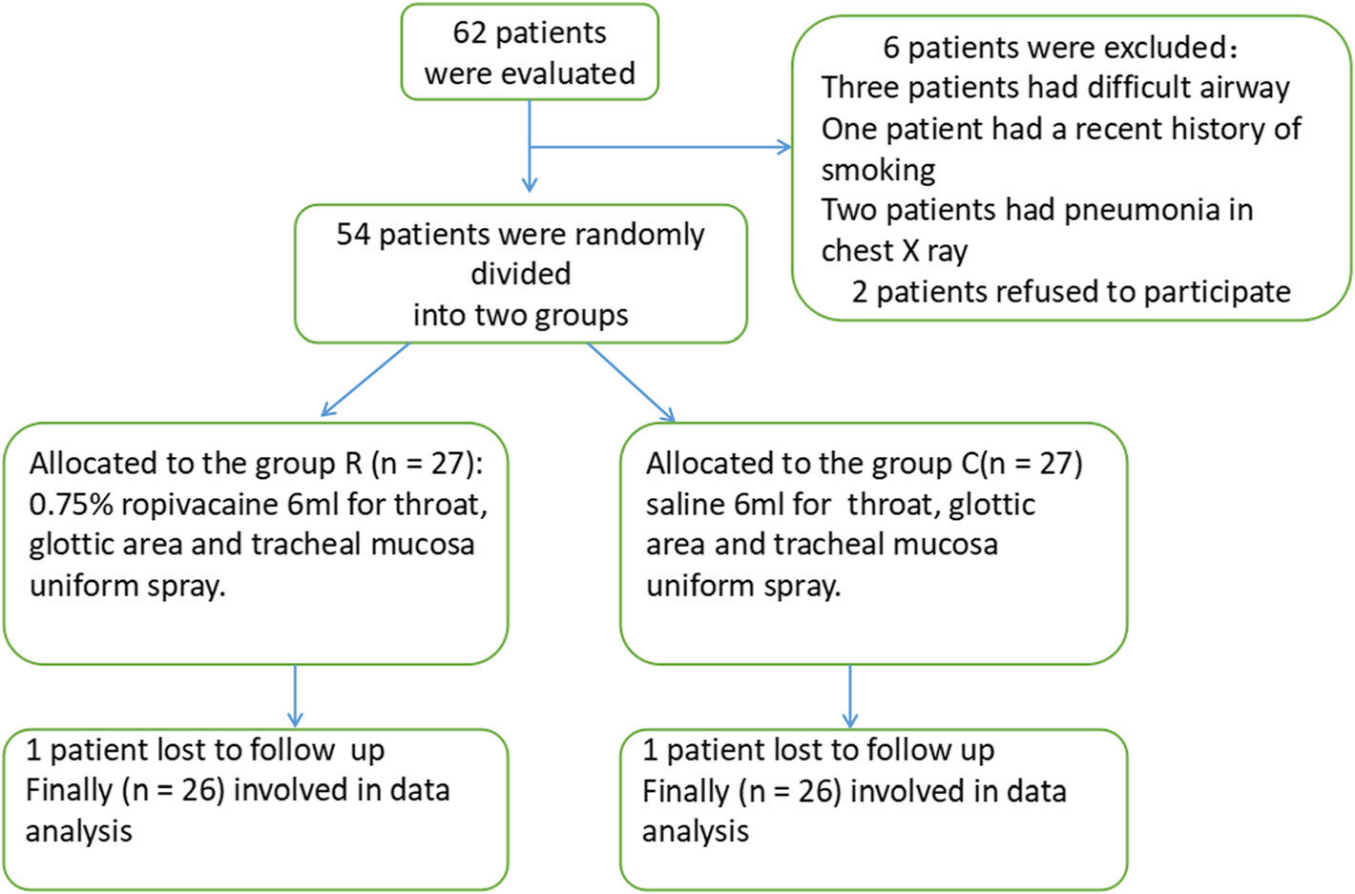
Consort flow chart that outline patients assignment and treatment protocols. Group R: the patients were recieved 6 ml of 0.75% ropivacaine for throat, glottic area and tracheal mucosa uniform spray; Group C: the patients were recieved with 6 ml of saline for throat, glottic area and tracheal mucosa uniform spray as control

A total of 62 patients were assessed for eligibility. Six patients did not meet the criteria, and two refused to participate in the study. In total, 54 patients were randomly assigned to Group C and Group R. However, two patients were lost to follow-up, and only 52 patients completed the study (Fig. 1). There were no significant differences in patient characteristics and operative procedures between the two groups (Table 1).

**Table 1:** Patients demographics and operative characteristics

	Group C	Group R	*P*
Age (years)	46.54 ± 12.93	49.54 ± 11.99	0.390
Gender:Male/Female	7/19	6/20	0.749
ASA classification (I/II)	7/19	9/17	0.548
Height (cm)	164.31 ± 6.20	165.08 ± 6.37	0.661
Weight (kg)	62.65 ± 10.74	66.38 ± 0.96	0.221
Operation time(min)	88.35 ± 36.46	104.54 ± 46.43	0.168
Anesthesia time (min)	107.42 ± 39.90	122.92 ± 48.52	0.214

Categorical variables were presented as numbers and percentage, quantitative variables were shown as means and standard deviation. *ASA* American Society of Anesthesiologists. Group R: the patients were recieved 6 ml of 0.75% ropivacaine for throat, glottic area and tracheal mucosa uniform spray; Group C: the patients were recieved with 6 ml of saline for throat, glottic area and tracheal mucosa uniform spray as control

**Fig. 2:**
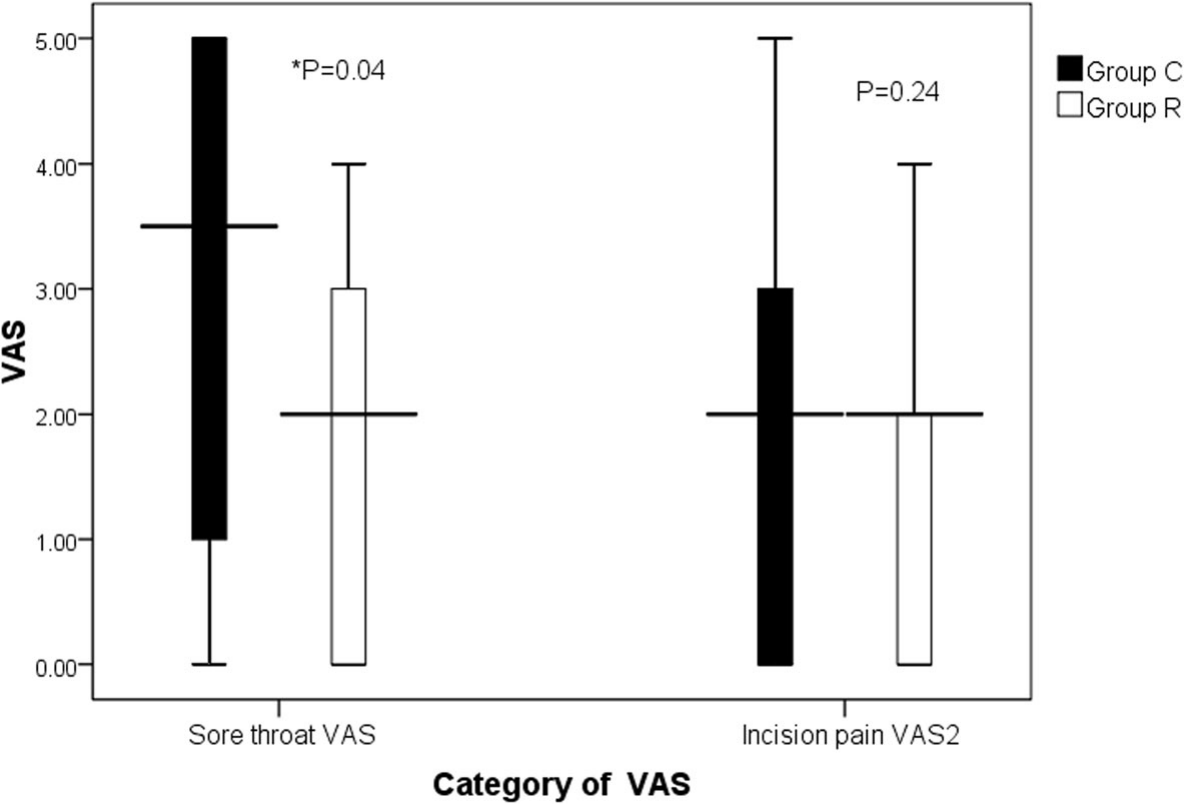
Incision pain scores (VAS). Data are presented as median (range). Group R: the patients were recieved 6 ml of 0.75% ropivacaine for throat, glottic area and tracheal mucosa uniform spray; Group C: the patients were recieved with 6 ml of saline for throat, glottic area and tracheal mucosa uniform spray as control.*P < 0.05 compared with group C

The cough rate in Group R was lower than that in Group C (34.62% vs. 76.92%, *P* = 0.002) (Table 2). As for patient recovery profiles, eye-opening time, extubation time, and PACU standing time showed no difference between the two groups. There was no significant difference between the two groups in the incidence of hypertension and agitation. There was no complications of nausea and vomiting (Table 3). The pharyngodynia score in Group R was lower than that in Group C at 12 h after operation (3.50 vs. 2.00, *P* = 0.040). No significant difference was found in 12 h incision pain score(Fig. 2). The patients in the two groups did not exhibit throat numbness, coughing during drinking, hoarseness and tone down.

**Table 2:** Overall cough rates and degree of cough

	Group R	Group C	*P*
The overall occurrence of cough	9(34.62%)	20(76.92%)	0.002^a^
Total number of patients according to cough grade			
Grade 0	17	6	0.002^a^
Grade 1	6	7	0.749
Grade 2	1	9	0.005^a^
Grade 3	2	4	0.358

Categorical variables were presented as numbers and percentage, quantitative variables were shown as means and standard deviation. *ASA* American Society of Anesthesiologists. Group R: the patients were recieved 6 ml of 0.75% ropivacaine for throat, glottic area and tracheal mucosa uniform spray; Group C: the patients were recieved with 6 ml of saline for throat, glottic area and tracheal mucosa uniform spray as control. ^a^ Compared GroupR with Group C, *P* < 0.05

**Table 3:** Recovery period profiles

	Group C	Group R	*P*
Eye-opening time (min)	14.92 ± 10.47	14.19 ± 9.96	0.798
Extubated time (min)	19.46 ± 10.08	18.69 ± 9.95	0.783
PACU standing time (min)	50.77 ± 15.07	49.69 ± 15.36	0.800
Agitation (number)	0	0	
Nausea and vomiting (number)	0	0	
Hypertension	4 (15.38%)	5 (19.23%)	0.500

Categorical variables were presented as numbers and percentage, quantitative variables were shown as means and standard deviation. ASA = American Society of Anesthesiologists. Group R: the patients were recieved 6 ml of 0.75% ropivacaine for throat, glottic area and tracheal mucosa uniform spray; Group C: the patients were recieved with 6 ml of saline for throat, glottic area and tracheal mucosa uniform spray as control

**Fig. 3:**
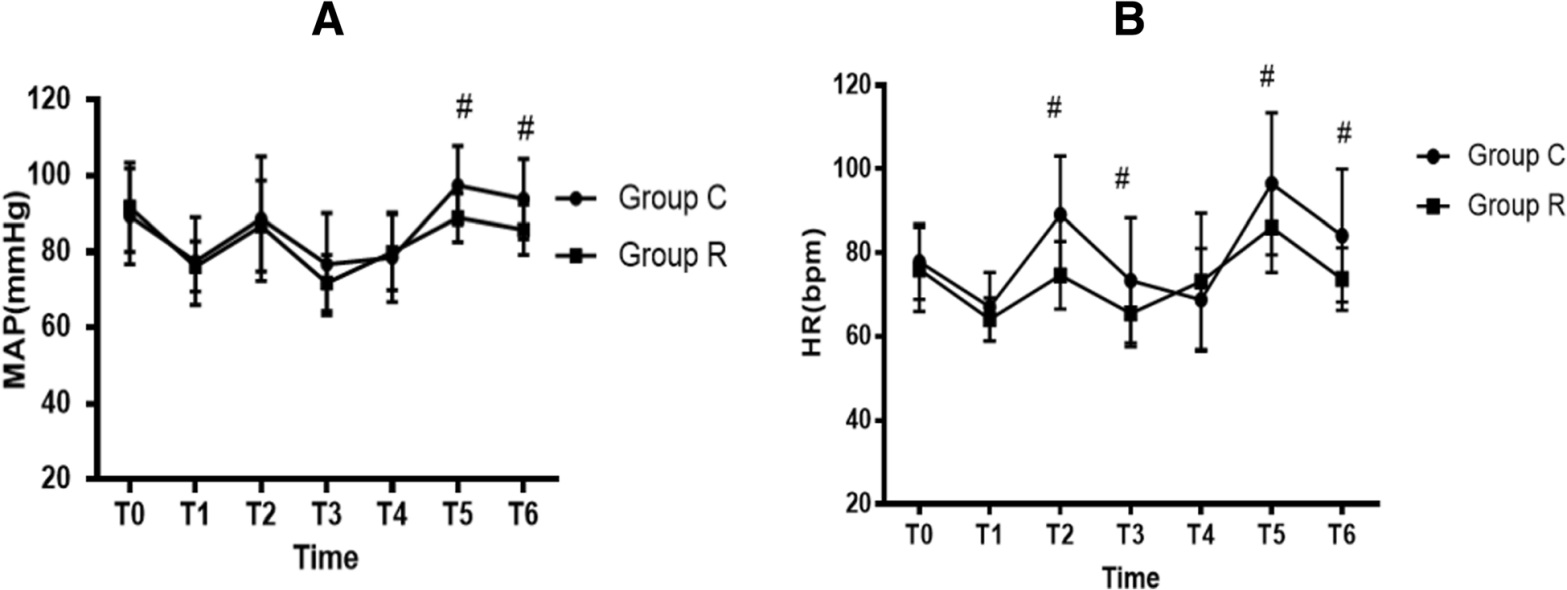
Hemodynamic values. **a:** MAP; **b:** HR. Data are presented as mean ± standard deviation Group R: the patients were recieved 6 ml of 0.75% ropivacaine for throat, glottic area and tracheal mucosa uniform spray; Group C: the patients were recieved with 6 ml of saline for throat, glottic area and tracheal mucosa uniform spray as control. HR = heart rate (beats/min); MAP = mean arterial blood pressure (mmHg); T_0_ = baseline (before anesthesia induction); T_1_ = before intubation; T_2_ = intubation immediately; T3 = 5 min after intubation; T4 = end of surgery; T5 = extubation immediately; T6 = 5 min after extubation. ^#^ *P* < 0.05 compared with group C. (MAP:T5#*P* = 0.001, MAP:T6#*P* = 0.002, HR:T2#*P* = 0.000, HR:T3#*P* = 0.023, HR:T5#*P* = 0.011, HR:T6#*P* = 0.004)

Hemodynamic values during surgery are shown in Fig. 3. Compared with Group C, MAP at T5 and T6 was significantly lower (Fig. 3a), and HR at T2, T3, T5, and T6 was obviously lower in Group R than in Group C (Fig. 3b).

## Discussion

The findings of this study showed that topical ropivacaine anesthesia before intubation could increase the tolerance to endotracheal tube. The incidence of cough during peri-extubation were decreased. In addition, it could also alleviate hemodynamic fluctuations during emergence and reduce the degree of sore throat after surgery.

Consist with previous researches [12, 13], the incidence of cough in Group R were significantly lower than those in Group C without respiratory depression, delayed awakening, and PONV. Ropivacaine, a kind of sodium channel blocker, suppresses cough by inhibiting the action potential formation in the tracheal touch-sensitive Aδ fibers (cough receptors) at specific concentrations [15]. Owing to its long action time, ropivacaine can maintain a valid blood concentration. Lidocaine is a medium-acting amide local anesthetic with short action time [16], thereby making it difficult to achieve relatively high concentrations during a long period time. Opioids may bring ahout possible respiratory depression, sedative effects, and PONV. Dexmedetomidine suppresses the airway reflex during extubation when the pantients without restoration of consciousness [17, 18].

Traditional spray surface anesthesia is limited to the throat and epiglottis. In this study, with the laryngo-tracheal mucosal atomization device (Wolfe Tory Medical, Inc. Produced in State of Utah, USA), we were able to apply the drugs at the throat, epiglottis and the trachea. Dense holes are distributed uniformly at the first 5 cm of the pipeline, which allows local anesthetics to be sprayed evenly, thereby enhancing the anesthetic effect. By blocking the sensitive area of the laryngeal nerve, recurrent laryngeal nerve, and glossopharyngeal nerve, cardiac conduction of the nerve impulse caused by stimulation of the tracheal catheter and sputum suctioning could be blocked [19].

Moreover, we found that patients’ throat pain after surgery was reduced by local ropivacaine. A systemic review showed that lidocaine can be used for preventing postoperative sore throat [20]. Similarly, ropivacaine can affect sensory-motor block and exhibits good liposolubility and long-acting effect [21]. It is widely used to control postoperative pain via intrathecal (lumbar) injection or local wound infiltration. Although it has low permeability, it can reach a valid blood concentration in a long time via mucous membrane.

Cervical hematoma is a common and dreaded complication in thyroid surgery [22]. In particular, cough during and after removal of the endotracheal tube may cause a ligature to slip or non-ligated small vessels to bleed profusely because of increased venous pressure. To reduce the risk of a fatal complication in thyroid surgery, it is important to achieve a smooth emergence. The recovery profile showed no difference between the two groups. We found no patient with agitation, nausea, and vomiting during the emergence and no patient with throat numbness at 12 h after surgery.

There were some limitations in our study. Firstly, the main limitation was that the optimum effective dose or concentration of local anesthetics was not determined. Secondly, our study only involved a single center. Thus, a multicenter study would be better in assuring the effectiveness of our anesthetic technique. Lastly, we merely observed the short-term effects of topical ropivacaine. The long-term outcomes and adverse effects need to be evaluated.

## Conclusion

In conclusion, this simple, fast, non-invasive method reduced the incidence of cough during peri-extubation without affecting patient recovery. Meanwhile it increased the tolerance to endotracheal tube with slight hemodynamic fluctuations and reduced the patients’ throat pain after thyroidectomy.

## Abbreviations

ASA: American Society of Anesthesiologists
DBP: Diastolic blood pressure
ERAS: Enhanced recovery after surgery
HR: Heart rate
ICU: Intensive care unit
MAP: Mean arterial pressure
PACU: Post-anesthesia care unit
PONV: Post Operative Nausea and Vomitting
SBP: Systolic blood pressure
SPO_2_: Blood oxygen saturation leve
VAS: Visual acuity scores
